# Supplementary material for: A general mechanism for initiating the bacterial general stress response
Source: eLife. 2025 Jun 6;13:RP100376. doi: 10.7554/eLife.100376 (PMC12143880; doi:10.7554/eLife.100376)

Figure 4 - Supplemental Figure 1 - source data 1: PDF file of original uncropped gel images of gels shown in Figure 4 - Supplemental Figure 1 B (top) and D (bottom).

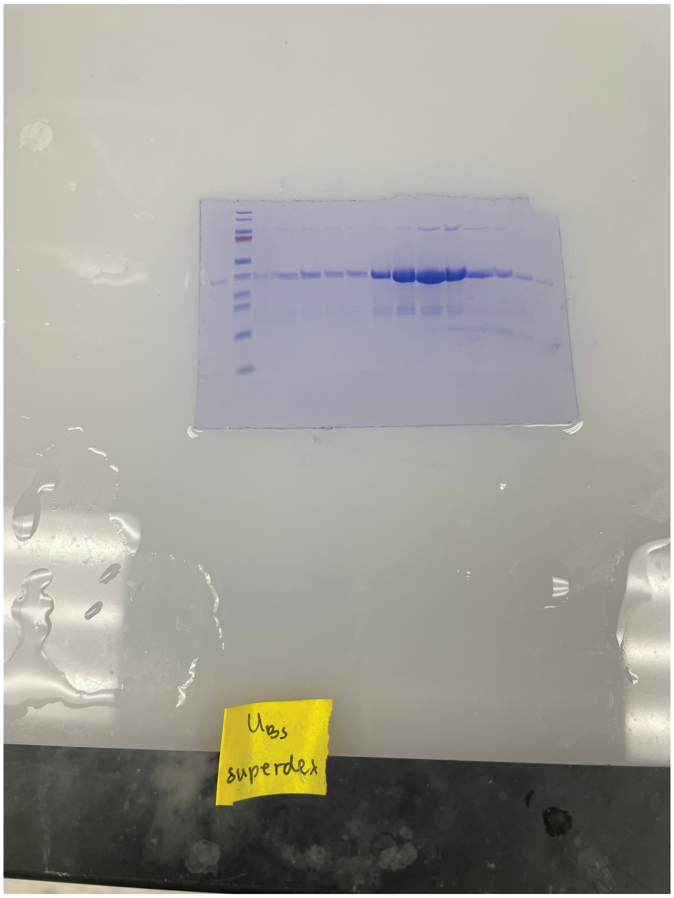

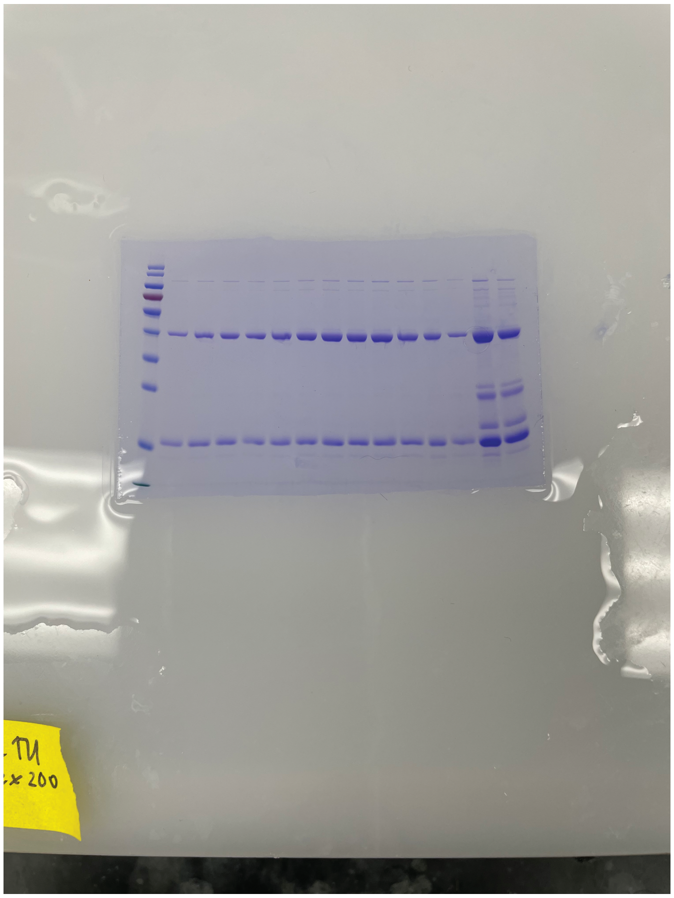

Supplement: Figure 4—figure supplement 1—source data 1. [file elife-100376-fig4-figsupp1-data1.pdf]
