# Supplementary figures and images for: A general mechanism for initiating the bacterial general stress response
Source: eLife. 2025 Jun 6;13:RP100376. doi: 10.7554/eLife.100376 (PMC12143880; doi:10.7554/eLife.100376)

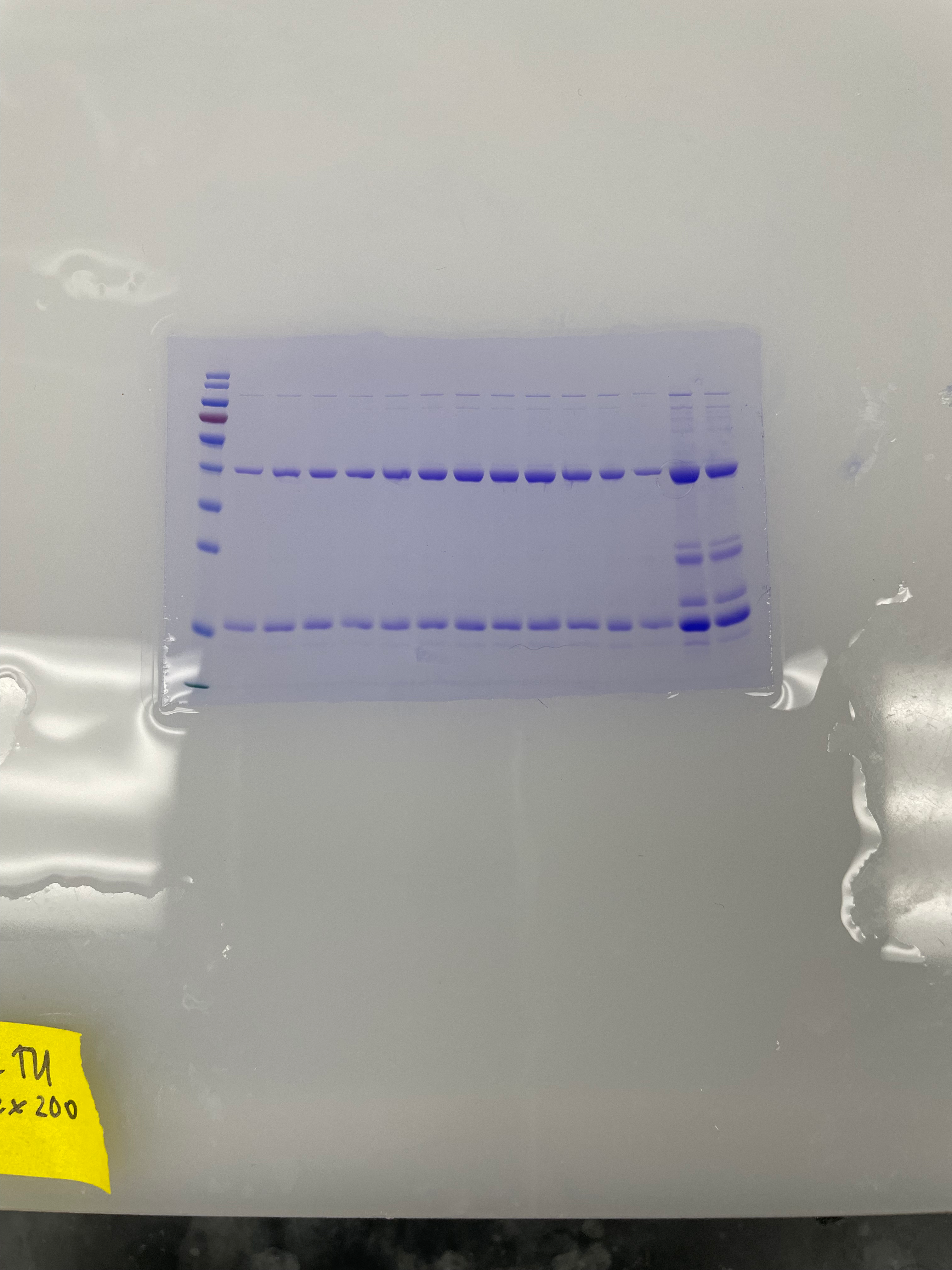

Supplement: Figure 4—figure supplement 1—source data 2. [file elife-100376-fig4-figsupp1-data2.zip › Figure 4 - Supplemental Figure 1-source data 2/Fig4Supp1D.png]

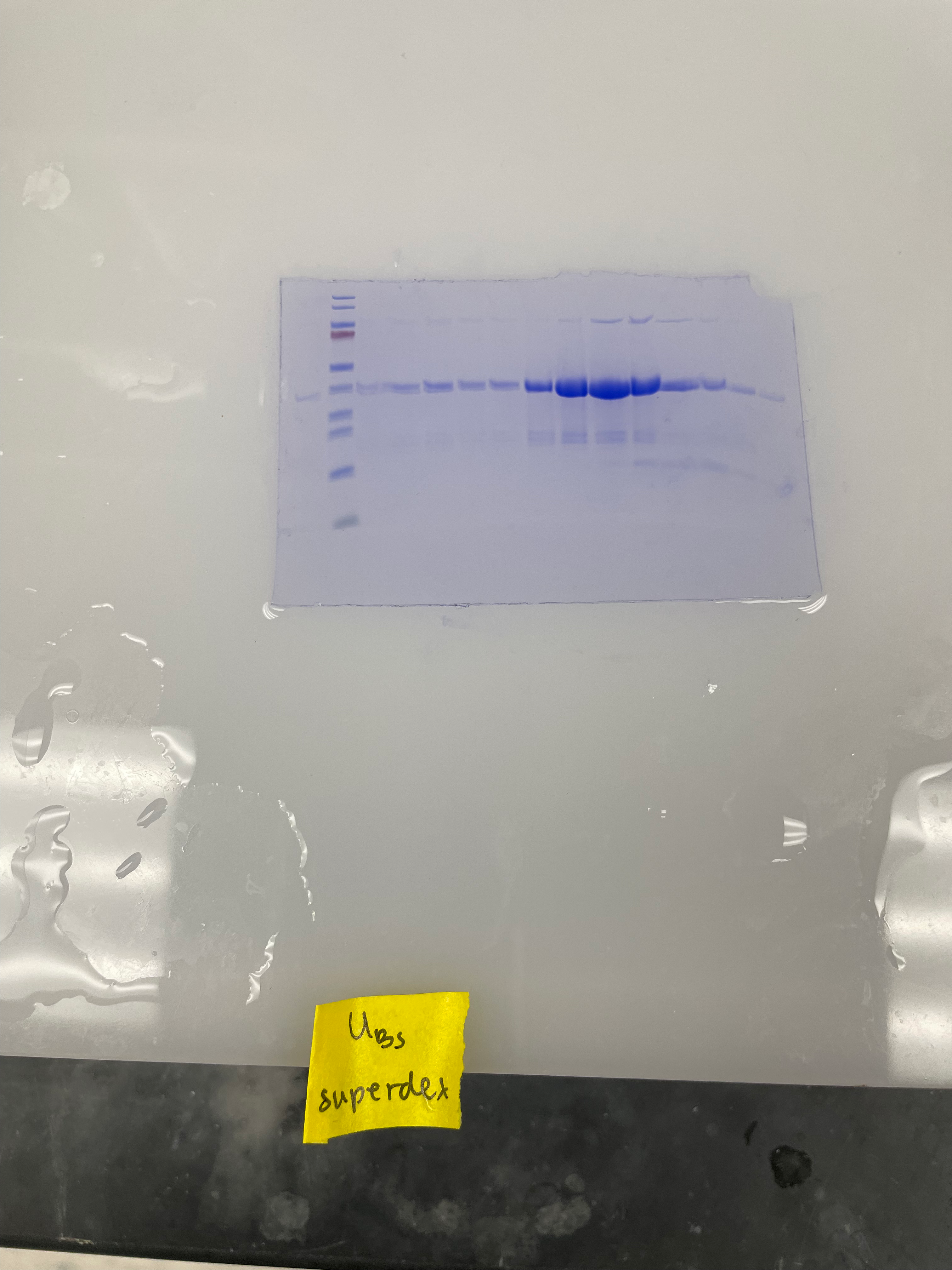

Supplement: Figure 4—figure supplement 1—source data 2. [file elife-100376-fig4-figsupp1-data2.zip › Figure 4 - Supplemental Figure 1-source data 2/Fig4Supp1B.png]
